# Suicide or undetermined intent? A register-based study of signs of misclassification

**DOI:** 10.1186/1478-7954-12-11

**Published:** 2014-04-17

**Authors:** Charlotte Björkenstam, Lars-Age Johansson, Peter Nordström, Ingemar Thiblin, Anna Fugelstad, Johan Hallqvist, Rickard Ljung

**Affiliations:** 1 Department of Public Health Sciences, Karolinska Institute, Stockholm, Sweden; 2 National Board of Health and Welfare, Stockholm, Sweden; 3 Department of Clinical Neuroscience, Karolinska Institutet, Stockholm, Sweden; 4 Deparment of Surgical Sciences, Uppsala University, Uppsala, Sweden; 5 Department of Public Health and Caring Sciences, Uppsala University, Uppsala, Sweden; 6 Institue of Environmental Medicine, Karolinska Institutet, Stockholm, Sweden

**Keywords:** Suicide, Undetermined intent, Unintentional poisonings, Mortality classification, Sweden, Register study, Psychotropics, Manner of death

## Abstract

**Background:**

Several studies have concluded that some deaths classified as undetermined intent are in fact suicides, and it is common in suicide research in Europe to include these deaths. Our aim was to investigate if information on background variables would be helpful in assessing if deaths classified as undetermined intent should be included in the analyses of suicides.

**Methods:**

We performed a register study of 31,883 deaths classified as suicides and 9,196 deaths classified as undetermined intent in Sweden from 1987 to 2011. We compared suicide deaths with deaths classified as undetermined intent with regard to different background variables such as sex, age, country of birth, marital status, prior inpatient care for self-inflicted harm, alcohol and drug abuse, psychiatric inpatient care, and use of psychotropics. We also performed a multivariate analysis with logistic regression.

**Results:**

Our results showed differences in most studied background factors. Higher education was more common in suicides; hospitalization for self-inflicted harm was more common among female suicides as was prior psychiatric inpatient care. Deaths in foreign-born men were classified as undetermined intent in a higher degree and hospitalization for substance abuse was more common in undetermined intents of both sexes. Roughly 50% of both suicide and deaths classified as undetermined intent had a filled prescription of psychotropics during their last six months. Our multivariate analysis showed male deaths to more likely be classified as suicide than female: OR: 1.13 (1.07-1.18). The probability of a death being classified as suicide was also increased for individuals aged 15–24, being born in Sweden, individuals who were married, and for deaths after 1987–1992.

**Conclusion:**

By analyzing Sweden’s unique high-validity population-based register data, we found several differences in background variables between deaths classified as suicide and deaths classified as undetermined intent. However, we were not able to clearly distinguish these two death manners. For future research we suggest, separate analyses of the two different manners of death.

## Introduction

Validation of national cause of death registers is warranted. These registers form the basis of healthcare planning on the community level; they are used for descriptive studies on mortality, follow-up studies of different diseases, and analytic studies on survival. Population-based register data are often powerful, useful tools, provided that each registration is complete and valid.

Suicide is a major public health problem. To target public health interventions and psychiatric care it is essential to be able to follow trends in suicide. Valid trends are dependent on correct classification of suicides. Before 1968 violent death was either classified as suicide, accident, or assault. This led to some degree of misclassification, as a physician had to establish the manner of death even when the information available was insufficient. Since the introduction of the eighth revision of the International Classification of Diseases (ICD) in 1968 it is possible to classify the manner of death as “undetermined intent”, which indicates that uncertainty exists as to the intention of death [1]. It has been suggested that suicide appears more susceptible than homicide and unintentional injury to misclassification under undetermined intent [2,3]. In the United States and Finland, the introduction of ICD-8 resulted in a 5-6% decrease in the reported number of suicides when deaths were transferred to the undetermined intent category [3]. Re-evaluation of the manner of death through additional information such as coroner’s records, family interviews, or hospital files has suggested that some deaths classified as undetermined intent are in fact suicides [4-11]. According to current guidelines, for a coroner or a forensic pathologist to establish suicide as the manner of death, there must be beyond reasonable doubt, otherwise the coroner is to classify manner of death as undetermined intent [7]. To allow comparisons between countries, the statistics used in the comparison must be based on an international standard. The definitions and instructions issued by the World Health Organization (WHO) in the ICD manuals are the only standard for mortality statistics that are universally accepted. WHO has provided detailed instructions for almost 60 years, and WHO member countries pledge to prepare mortality statistics according to these specifications [12]. Accordingly, Sweden follows these international rules for classifying causes of death. There are continuous up-dates on these regulations. Between ICD-9 and ICD-10, there were profound changes in the rules of classifying unintentional poisonings. Other changes in the rules of classification affected how to select the underlying cause of death regarding unintentional poisoning. However, due to social and cultural norms death certification practices can vary over time and between countries and regions. It can also vary between different coroners at the same department. Hence, comparisons over time and between regions can be difficult since the inclination to select suicide might differ.

One study in California stated that it might be unadvisable to use official suicide data in scientific studies unless one estimates and corrects for underreporting of suicides [9]. So far there is no agreement on the amount to be corrected for. Some claim the official suicide *trends* are accurate despite the alleged underreporting of suicides [13].

Another possible misclassification is suicides classified as unintentional injuries, foremost unintentional poisonings. According to an American study the recent official US decline in suicide rates was to some extent due to an artifact of misclassifying nonelderly suicides as unintentional poisonings [14]. Previously, another American study found that several known risk factors for suicide were more frequently reported as present in unintentional poisoning deaths than in deaths coded as suicide poisonings [15]. Suicide case ascertainment of the deceased with a history of concomitant alcohol and other substance abuse can be complicated as they are risk factors for suicide but also can diminish the likelihood of a coroner establishing death as a result of suicide, instead classifying death as due to alcohol or drugs [16,17].

In this study we investigate trends and systematic differences in background information between deaths classified as suicide and deaths classified as undetermined intent from 1987 to 2011 in Sweden and for poisonings, including unintentional poisoning, from 1997 to 2011, to assess their implications for the misclassification of suicide. Since a majority of deaths classified as undetermined intent are poisonings, and it is often difficult to establish intent in these deaths, we chose to study poisonings separately in a subanalysis. Our aim was to investigate if information on background variables would be helpful in assessing if deaths classified as undetermined intent should be included in analyses of suicides.

## Materials and methods

### Ethics statement

The study population was based on linkage of several public national registers. Ethical vetting is always required when using register data in Sweden. The ethical vetting is performed by regional ethical review boards and the risk appraisal associated with the Law on Public Disclosure and Secrecy is done by data owners. The ethical review boards can however waive the requirement to consult the data subjects (or in case of minors/children the next of kin, careers, or guardians) directly to obtain their informed consent, and will often do so if the research is supported by the ethical review board and the data have already been collected in some other context. According to these standards in Sweden, this project has been evaluated and approved by the Regional Ethical Review Board of Karolinska Institutet, Stockholm, Sweden.

### Study population

We selected all deaths in Sweden between 1987 and 2011 classified as suicide.

(ICD-9: E950-E959, ICD-10: X60-X84) and deaths classified as undetermined intent (ICD-9: E980-E989, ICD-10: Y10-Y34) from the Causes of Death Register. ICD defines undetermined intent as “events where available information is insufficient to enable a medical or legal authority to make a distinction between accident, self-harm and assault” [12]. No changes in the rules of classification occurred during the time period studied. Since a majority of deaths classified as undetermined intent are poisonings, and it is often difficult to establish intent in these deaths, we chose to study poisonings separately in a subanalysis, also including unintentional poisonings (ICD-10: X40-X49). All poisonings regardless of substance (legal or illegal) were included.

We chose background variables in accordance to relevance and also what is available in the routinely collected registers we were able to access. We retrieved information on age, sex, country of birth, marital status, highest attained educational level, history of mental illness, self-inflicted harm, hospitalization for substance abuse, and use of psychotropic drugs to categorize each death. The unique personal identity number assigned to each Swedish resident was used to link together information from the different population-based registers.

### Registers

Sweden and the other Nordic countries have a long tradition of collecting data on diseases and deaths. We employ epidemiological registers of high quality, covering the whole population, and some reach back to the 1950s [12]. The Causes of Death Register contains information on deceased Swedish residents since 1952. Since 1997 all deceased are included (i.e., even those where a death certificate never was sent in), though for around 2% there is a lack of medical information. Every year the forensic departments receive more than 5,000 deceased for whom they are to establish the cause of death and whether or not it was due to assault. Medical departments within the Swedish healthcare service do not perform these autopsies; all suspected suicides should be examined at the forensic department, and 98% are. The autopsy proportion of deaths classified as undetermined intent is similar and it is slightly higher than 90% for deaths classified as unintentional poisonings.

The National Patient Register contains information on all inpatient care in Sweden since 1987, although information on psychiatric care is available since 1973 [18].

The Swedish Prescribed Drug Register contains information on all prescribed drugs dispensed at a pharmacy for the entire population of Sweden since July 2005 and includes personal identity number [19].

We used educational level selected from the Swedish Register of Education held by Statistics Sweden as measure of social stratification. This information was only available between 1997 and 2010. Information on age, sex, country of birth, and marital status is available in the Cause of Death Register.

### Categorization of background information for each death

#### Age

We classified all deaths according to age into the following categories: 15–24, 25–44, 45–64, and 65 + .

#### Method of suicide

We categorized both suicides and undetermined intents into seven subcategories according to the chosen method: poisoning, hanging, drowning, jumping, cutting, firearms, and other.

#### Country of birth

All individuals were classified as either born in Sweden or foreign-born.

#### Marital status

Marital status was categorized at time of death into four groups: married, widowed, divorced, and unmarried.

#### Educational level

Level of education was accessible between 1997 and 2010 and was obtained from the Swedish Register of Education at Statistics Sweden. We classified educational level into three categories: 1: nine-year (compulsory school), 2: 10–12 year education (senior high school), 3: >12 years (university education).

#### Hospitalization

We obtained information on hospitalization in psychiatric in-patient care (hospitalization for substance abuse was studied separately, see below), within one and five years prior to death. Hospitalization for self-inflicted harm (ICD-9: E950-E959, ICD-10: X60-X84) and substance abuse (ICD-9: 291, 303, 305.0, 357.5, 425.5, 535.3, 571.0-571.3, E860, E980 + N980, 304, 305.0-305.7, 305.9, 965.0, 968.5, 969.6, 969.7 and ICD-10: F11, F12, F13, F14, F15, F16, F18, F19, O355, P044, E244, F10, G312, G621, G721, I426, K292, K700-K709, K852, K860, O354, P043, Q860, T510-T519, Y901-Y909, Y911-Y919, Z502, Z714, Z721) were also studied separately.

All psychiatric care including care for substance abuse is also analyzed over time.

#### Psychotropic medication

From the Prescribed Drug Register we selected dispensed prescriptions of psychotropic medications identified by their specific Anatomical Therapeutic Chemical (ATC) codes (N05A neuroleptics, N05B sedatives, N05C soporifics and sedatives, and N06AB SSRI, and N06A other antidepressants) between 2006 and 2010. Psychotropic medication use was defined as “current”, “recent”, “past”, or “former” if the drug had been dispensed 0–30 days, 31–180 days, 181–365 days, or 1–2 years before the date of death for each specific drug, respectively. The absence of a prescription for psychotropic medication was classified as “non-use”. Percentage with a prescription is presented.

### Analyses and presentation of results

We present cohort characteristics calculated as percentage with 95% confidence intervals to compare the distribution of different characteristics between different manners of death. We also calculate and compare ratios of deaths with undetermined intent to suicide between categories of different background variables. We present trends of age-standardized death rates per 100,000 inhabitants. A ratio of 1 equals the same number of suicides as undetermined intents, whereas a ratio less than 1 equals fewer undetermined intents, and a ratio above 1 equals more undetermined intents than suicides.

#### Ratio = undetermined intents/suicides

Trends of age-standardized death rates per 100,000 inhabitants are also presented. Analyses of educational level were restricted to individuals aged 35–74.

To analyze the demographic background variables we also performed a multivariate analysis with mode of death, suicide or undetermined intent, as the dependent variable.

## Results

### Background information

In total 46,909 deaths were under study, with 31,883 classified as suicides in 1987–2011, 9,196 classified as deaths with undetermined intent in 1987–2011, and 5,830 classified as unintentional poisonings in 1997–2011. Characteristics of the studied deaths are presented in Table 1. Divorce was more frequent among deaths classified as undetermined intent, 27.7% [27.7-28.6], compared to suicides, 19.9% [19.4-20.3]. A higher proportion of suicides had university education, 17.6% [17.0-18.2], compared to deaths classified as undetermined intent, 13.0% [11.9-14.0]. Women committing suicide were more likely to have previously been hospitalized for self-inflicted harm. Earlier psychiatric hospitalization was more common among female suicides, 38.1% [36.9-39.3], compared to 26.7% [24.8-28.6] in deaths of undetermined intent. No statistically significant difference was seen in men for psychiatric hospitalization over the previous five years. Hospitalization for substance abuse was most common among men with deaths classified as undetermined intent.

**Table 1:** Cohort characteristics of suicides and undetermined intents, 1987–2011, percentage with 95% CI, by sex

	**Women**		**Men**		**Total**	
	**Suicide**	**Undetermined**	**Suicide**	**Undetermined**	**Suicide**	**Undetermined**
Marital status	9 381	2 909	22 502	6 287	31 883	9 196
Married	27.0 (26.1-27.9)	24.3 (22.8-25.9)	30.2 (29.6-30.8)	16.2 (15.3-17.1)	29.3 (28.8-29.8)	18.8 (18.0-19.6)
Divorced	24.5 (23.6-25.4)	31.7 (30.0-33.4)	17.9 (17.4-18.4)	25.8 (24.7-26.9)	19.9 (19.4-20.3)	27.7 (26.7-28.6)
Unmarried	32.6 (31.7-33.6)	28.9 (27.3-30.6)	44.2 (43.6-44.9)	53.3 (52.0-54.5)	40.8 (40.3-41.3)	45.6 (44.6-46.6)
Widowed	15.8 (15.1-16.5)	15.1 (13.8-16.4)	7.3 (6.9-7.6)	4.6 (4.1-5.2)	9.8 (9.5-10.1)	7.9 (7.4-8.5)
Born outside Sweden	9 381	2 909	22 502	6 287	31 883	9 196
Yes	14.4 (13.7-15.1)	15.4 (14.1-16.7)	11.1 (10.7-11.5)	15.1 (14.3-16.0)	12.1 (11.7-12.4)	15.2 (14.5-16.0)
No	85.6 (84.9-86.3)	84.6 (83.3-85.9)	88.9 (88.5-89.3)	84.9 (84.0-85.7)	87.9 (87.6-88.3)	84.8 (84.0-85.5)
Education*	4 543	1 283	11 304	2 752	15 847	4 035
−9 years	34.5 (33.1-35.9)	39.4 (36.8-42.1)	39.9 (39.0-40.8)	41.4 (39.5-43.2)	38.4 (37.6-39.1)	40.7 (39.2-42.3)
9-12 years	43.4 (42.0-44.9)	43.7 (41.0-46.4)	44.3 (43.4-45.2)	47.5 (45.6-49.3)	44.0 (43.3-44.8)	46.3 (44.8-47.8)
>12 years	22.1 (20.9-23.3)	16.8 (14.8-18.9)	15.8 (15.1-16.5)	11.2 (10.0-12.3)	17.6 (17.0-18.2)	13.0 (11.9-14.0)
Hospitalization self-inflicted harm**	6 797	2 014	16 368	4 397	23 165	6 411
1 year	11.2 (10.5-11.9)	5.9 (4.9-6.9)	5.1 (4.8-5.4)	3.9 (3.3-4.5)	6.9 (6.6-7.2)	4.5 (4.0-5.0)
3 years	18.1 (17.2-19.0)	11 (9.6-12.4)	8.2 (7.8-8.6)	7.1 (6.3-7.9)	11.1 (10.7-11.5)	8.3 (7.6-9.0)
5 years	21.1 (20.1-22.1)	14.3 (12.8-15.8)	9.6 (9.1-10.1)	9.2 (8.3-10.1)	13.0 (12.6-13.4)	10.8 (10–11.6)
None	49.6 (48.4-50.8)	68.9 (66.9-70.9)	77.2 (76.6-77.8)	79.8 (78.6-81)	69.1 (68.5-69.7)	76.4 (75.4-77.4)
Psychiatric hospitalization**	6 797	2 014	16 368	4 397	23 165	6 411
1 year	25.5 (24.5-26.5)	13.2 (11.7-14.7)	14.2 (13.7-14.7)	9.8 (8.9-10.7)	17.5 (17.0-18.0)	10.9 (10.1-11.7)
3 years	34.0 (32.9-35.1)	22.3 (20.5-24.1)	19.6 (19.0-20.2)	16.5 (15.4-17.6)	23.8 (23.3-24.3)	18.3 (17.4-19.2)
5 years	38.1 (36.9-39.3)	26.7 (24.8-28.6)	22.2 (21.6-22.8)	20.7 (19.5-21.9)	26.9 (26.3-27.5)	22.6 (21.6-23.6)
None	2.4 (2.0-2.8)	37.8 (35.7-39.9)	44.0 (43.2-44.8)	53.0 (51.5-54.5)	31.8 (31.2-32.4)	48.2 (47.0-49.4)
Hospitalization abuse**	6 797	2 014	16 368	4 397	23 165	6 411
1 year	4.5 (4.0-5.0)	10.5 (9.2-11.8)	5.5 (5.2-5.8)	15.6 (14.5-16.7)	5.2 (4.9-5.5)	14.0 (13.2-14.8)
3 years	7.3 (6.7-7.9)	16.8 (15.2-18.4)	8.4 (8.0-8.8)	25.1 (23.8-26.4)	8.1 (7.7-8.5)	22.5 (21.5-23.5)
5 years	8.8 (8.1-9.5)	20.7 (18.9-22.5)	10.0 (9.5-10.5)	30.0 (28.6-31.4)	9.6 (9.2-10.0)	27.1 (26.0-28.2)
None	79.4 (78.4-80.4)	52.0 (49.8-54.2)	76.1 (75.4-76.8)	29.3 (28.0-30.6)	77.1 (76.6-77.6)	36.4 (35.2-37.6)

*Only available 1997-2010.

**Both alcohol and other drugs.

**Figure 1:**
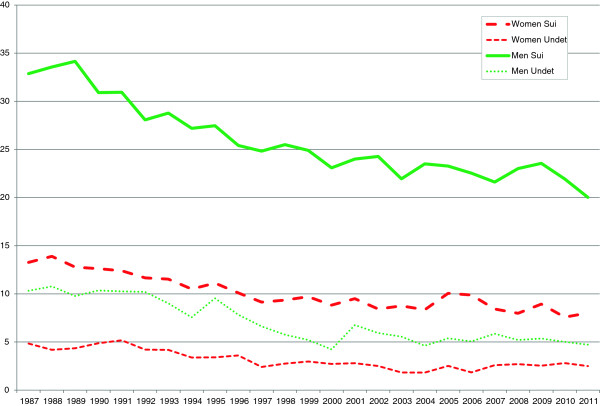
Age standardized mortality rates per 100 000 inhabitants of suicide and undetermined intent 1987-2011, by sex.

From 1987 to 2011 deaths classified as suicides decreased from 13.3 per 100,000 to 8.0 per 100,000 in women and from 32.9 to 20.0 per 100,000 in men (Figure 1).

Deaths classified as undetermined intent decreased from 1987 to 2011, in women from 4.8 to 2.5 per 100,000, and in men from 10.3 to 4.7 per 100,000.

### Death method used

Table 2 displays the number of deaths due to method, sex, and age group. Poisoning was the most frequent method among deaths classified as undetermined intent for all age groups and in both men and women. Poisoning was the most frequent method among female suicides except in the youngest age group where hanging was most common. Hanging was the most frequent method among male suicides in all ages. The number of poisonings decreased during the studied years, among both those coded as suicide (7.8 to 3.5 per 100,000) and undetermined intent (4.5 to 2.5 per 100,000).

**Table 2:** Number of deaths with row percentages, 1987-2011, by method, sex, and age group.

**Age group**	**Suicide**							**Undetermined**						
**Women**	**Poisoning**	**Hanging**	**Drowning**	**Jump**	**Cutting**	**Firearms**	**Other**	**Poisoning**	**Hanging**	**Drowning**	**Jump**	**Cutting**	**Firearms**	**Other**
15-24	239 (29%)	332 (41%)	30 (4%)	58 (7%)	5 (1%)	17 (2%)	134 (16%)	89 (55%)	4 (2%)	15 (9%)	13 (8%)	1 (1%)	4 (2%)	35 (22%)
25-44	1 236 (46%)	769 (29%)	147 (6%)	170 (6%)	47 (2%)	36 (1%)	263 (10%)	545 (73%)	10 (1%)	70 (9%)	19 (3%)	5 (1%)	6 (1%)	90 (12%)
45-64	1 648 (49%)	757 (22%)	421 (13%)	183 (5%)	69 (2%)	26 (1%)	262 (9%)	963 (75%)	11 (1%)	160 (12%)	26 (2%)	6 (0%)	2 (0%)	122 (9%)
65-	1 021 (40%)	563 (22%)	543 (21%)	245 (10%)	44 (2%)	8 (0%)	108 (4%)	375 (53%)	9 (1%)	192 (27%)	43 (6%)	3 (0%)	1 (0%)	90 (13%)
All	4 144 (44%)	2 421 (26%)	1 141 (12%)	656 (7%)	165 (2%)	87 (1%)	767 (8%)	1 972 (68%)	34 (1%)	437 (15%)	101 (3%)	15 (1%)	13 (0%)	337 (12%)
Men														
15-24	366 (19%)	841 (43%)	35 (2%)	122 (6%)	19 (1%)	316 (16%)	279 (14%)	255 (48%)	16 (3%)	69 (13%)	33 (6%)	8 (1%)	17 (3%)	138 (26%)
25-44	2 063 (30%)	2 644 (38%)	206 (3%)	319 (5%)	209 (3%)	892 (13%)	593 (9%)	1 525 (67%)	33 (1%)	221 (10%)	87 (4%)	19 (1%)	32 (1%)	358 (16%)
45-64	1 988 (26%)	3 082 (40%)	318 (4%)	254 (3%)	278 (4%)	1 342 (17%)	532 (7%)	1 568 (64%)	23 (1%)	342 (14%)	67 (3%)	13 (1%)	20 (1%)	418 (17%)
65-	970 (17%)	2 377 (41%)	457 (8%)	352 (6%)	215 (4%)	1 151 (20%)	223 (4%)	437 (43%)	11 (1%)	277 (27%)	79 (8%)	3 (0%)	6 (1%)	212 (21%)
All	5 387 (24%)	8 944 (40%)	1 016 (5%)	1 047 (5%)	721 (3%)	3 701 (16%)	1 627 (7%)	3 785 (60%)	83 (1%)	909 (14%)	266 (4%)	43 (1%)	75 (1%)	1 126 (18%)
Total														
15-24	605 (22%)	1 173 (42%)	65 (2%)	180 (6%)	24 (1%)	333 (12%)	413 (15%)	344 (49%)	20 (3%)	84 (12%)	46 (7%)	9 (1%)	21 (3%)	173 (25%)
25-44	3 299 (34%)	3 413 (36%)	353 (4%)	489 (5%)	256 (3%)	928 (10%)	856 (9%)	2 070 (69%)	43 (1%)	291 (10%)	106 (4%)	24 (1%)	38 (1%)	448 (15%)
45-64	3 636 (33%)	3 839 (34%)	739 (7%)	437 (4%)	347 (3%)	1 368 (12%)	794 (7%)	2 531 (68%)	34 (1%)	502 (13%)	93 (2%)	19 (1%)	22 (1%)	540 (14%)
65-	1 991 (24%)	2 940 (35%)	1 140 (14%)	597 (7%)	259 (3%)	1 159 (14%)	331 (4%)	812 (47%)	20 (1%)	469 (27%)	122 (7%)	6 (0%)	7 (0%)	302 (17%)
All	9 531 (30%)	11 365 (36%)	2 157 (7%)	1 703 (5%)	886 (2%)	3 788 (12%)	2 394 (8%)	5 757 (63%)	117 (1%)	1 346 (15%)	367 (4%)	58 (1%)	88 (1%)	1 463 (16%)

### Hospitalization

**Figure 2:**
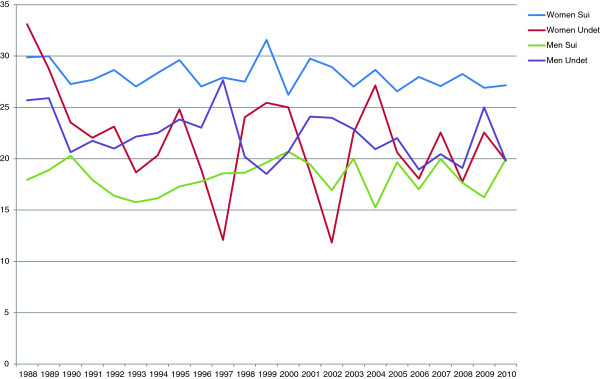
Percentage hospitalized for psychiatric care during the last year, 1988-2010.

**Figure 3:**
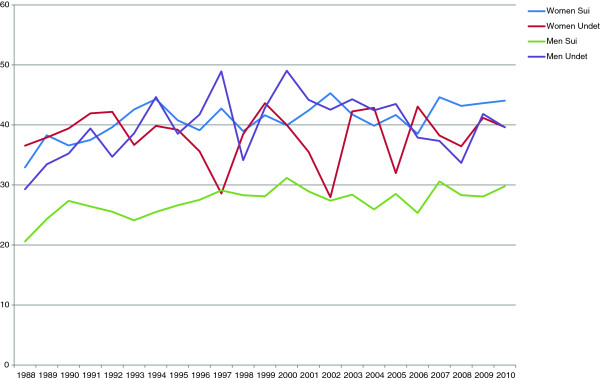
Percentage hospitalized for psychiatric care during the last five years, 1988-2010.

Among women who later committed suicide, close to 30% had been treated in psychiatric inpatient care during their last year prior to suicide (Figure 2). For deaths classified as undetermined intent the percentage was lower. Among male suicides, fewer than 20% had been treated in psychiatric inpatient care in the year prior to suicide, compared to 20-25% of male deaths coded as undetermined intent. Around 35% of both male and female deaths coded as undetermined intent, 35% of female suicides, and 25% of male suicides had been treated in psychiatric inpatient care at some point in the five years prior to death (Figure 3).

### Poisonings including unintentional manner

Table 3 displays cohort characteristics for poisoning classified as suicide, undetermined intent, and unintentional manner. Regardless of the manner of death, poisonings were most common in the age group of 45–64 years. Married status was more common among male suicides, as was widowhood in both sexes. Unintentional poisonings were more common among foreign-born males. Longer education was also more common in suicides in both sexes. Earlier hospitalization for self-inflicted harm was more common among both female deaths classified as suicides 22.1% [20.4-23.8], compared to 16.1% [14.0-18.2] among undetermined intent, and 8.3% [6.9-9.7] among unintentional poisonings as well as among male suicides 15.4% [14.0-16.8], compared to 10.9% [9.6-12.2] among undetermined intent, and 7.3% [6.5-8.1] among unintentional. Former psychiatric inpatient care showed a similar gradient, being more common among suicides. Hospitalization for substance abuse (alcohol included) was most common among deaths classified as undetermined intent, followed by unintentional poisonings, and least common among suicides, regardless of sex.

**Table 3:** Cohort characteristics of poisonings as suicides, undetermined intents, and unintentional, percentage with 96% CI, 1997–2011, by sex

	**Women**			**Men**		
***Marital Status***	**Suicide**	**Undetermined**	**Unintentional**	**Suicide**	**Undetermined**	**Unintentional**
Married	20.9 (19.3-22.5)	20.9 (18.5-23.3)	20.1 (18.1-22.1)	22.6 (21.0-24.2)	10.1 (8.9-11.3)	12.0 (11.0-13.0)
Divorced	32.5 (30.6-34.4)	36.5 (33.7-39.3)	31.4 (29.1-33.7)	25.9 (24.3-27.5)	27.0 (25.2-28.8)	25.6 (24.3-26.9)
Unmarried	31.0 (29.1-32.9)	31.9 (29.2-34.6)	28.1 (25.8-30.4)	44.8 (42.9-46.7)	59.9 (57.9-61.9)	58.1 (56.6-59.6)
Widowed	15.5 (14.0-17.0)	10.8 (9.0-12.6)	20.3 (18.3-22.3)	6.8 (5.9-7.7)	2.9 (2.2-3.6)	4.3 (3.7-4.9)
*Born outside Sweden*						
Yes	15.4 (13.9-16.9)	14.2 (12.2-16.2)	15.1 (13.3-16.9)	12.7 (11.5-13.9)	12.4 (11.0-13.8)	15.4 (14.3-16.5)
No	84.6 (83.1-86.1)	85.8 (83.8-87.8)	85.1 (83.3-86.9)	87.3 (86.1-88.5)	87.6 (86.2-89.0)	84.6 (83.5-85.7)
Education*						
−9 years	27.2 (24.9-29.5)	34.9 (31.4-38.4)	31.4 (27.6-35.2)	31.6 (29.4-33.8)	37.6 (34.9-40.3)	40.2 (38.0-42.4)
9-12 years	48.5 (45.9-51.1)	48.3 (44.7-51.9)	52.2 (48.1-56.3)	50.3 (47.9-52.7)	50.3 (47.5-53.1)	50.3 (48.1-52.5)
>12 years	24.3 (22.1-26.5)	16.8 (14.1-19.5)	16.4 (13.3-19.5)	18.1 (16.3-19.9)	12.2 (10.4-14.0)	9.6 (8.3-10.9)
Hospitalization self-inflicted harm**						
1 year	10.1 (8.9-11.3)	6.5 (5.1-7.9)	2.7 (1.9-3.5)	7.9 (6.9-8.9)	4.5 (3.6-5.4)	2.4 (1.9-2.9)
3 years	18.0 (16.4-19.6)	11.9 (10.0-13.8)	6.7 (5.4-8.0)	13.0 (11.7-14.3)	8.6 (7.4-9.8)	5.6 (4.9-6.3)
5 years	22.1 (20.4-23.8)	16.1 (14.0-18.2)	8.3 (6.9-9.7)	15.4 (14.0-16.8)	10.9 (9.6-12.2)	7.3 (6.5-8.1)
None	49.8 (47.8-51.8)	65.5 (62.7-68.3)	82.2 (80.3-84.1)	63.8 (62.0-65.6)	75.9 (74.1-77.7)	84.7 (83.6-85.8)
Psychiatric hospitalization**						
1 year	20.9 (19.3-22.5)	12.1 (10.2-14.0)	5.3 (4.2-6.4)	15.4 (14.0-16.8)	9.1 (7.9-10.3)	5.3 (4.6-6.0)
3 years	30.1 (28.2-32.0)	20.4 (18.1-22.7)	9.8 (8.3-11.3)	22.5 (20.9-24.1)	17.8 (16.2-19.4)	10.5 (9.6-11.4)
5 years	35.0 (33.1-36.9)	25.3 (22.8-27.8)	13.4 (11.7-15.1)	26.1 (24.5-27.7)	21.8 (20.1-23.5)	13.8 (12.8-14.8)
None	14.0 (12.6-15.4)	42.2 (39.3-45.1)	71.5 (69.2-73.8)	36.0 (34.2-37.8)	51.3 (49.2-53.4)	70.3 (68.9-71.7)
Hospitalization abuse**						
1 year	5.2 (4.3-6.1)	12.1 (10.2-14.0)	8.0 (6.6-9.4)	8.0 (7.0-9.0)	16.4 (14.9-17.9)	14.5 (13.5-15.5)
3 years	9.0 (7.8-10.2)	19.2 (16.9-21.5)	14.0 (12.2-15.8)	13.2 (11.9-14.5)	27.3 (25.5-29.1)	24.1 (22.8-25.4)
5 years	10.9 (9.6-12.2)	22.7 (20.3-25.1)	17.4 (15.5-19.3)	15.7 (14.3-17.1)	32.6 (30.7-34.5)	28.8 (27.5-30.1)
None	74.9 (73.1-76.7)	45.9 (43.0-48.8)	60.6 (58.1-63.1)	63.2 (61.4-65.0)	23.7 (22.0-25.4)	32.6 (31.2-34.0)
Age groups						
15-24	5.6 (4.6-6.6)	5.4 (4.0-6.8)	5.3 (4.2-6.4)	5.0 (4.2-5.8)	9.8 (8.5-11.1)	8.9 (8.1-9.7)
25-44	27.5 (25.6-29.4)	24.3 (21.6-27.0)	17.9 (16.0-19.8)	33.5 (31.7-35.3)	38.6 (36.4-40.8)	32.5 (31.1-33.9)
45-64	42.1 (40.0-44.2)	52.3 (49.2-55.4)	41.8 (39.3-44.3)	41.7 (39.8-43.6)	40.8 (38.6-43.0)	41.9 (40.4-43.4)

*Only available 1997-2010.

**Both alcohol and other drugs.

The number of deaths per 100,000 in both women and men due to suicide poisonings and deaths by poisoning classified as undetermined intent slightly decreased during the study period, whereas unintentional poisonings showed an incline from around year (data not shown).

The ratio of undetermined intent poisonings to all suicide poisonings was lowest among those with university education (data not shown).

### Dispensed prescribed drugs

Among the 5,870 suicides in 2006 to 2012 (75.1% among women and 60.0% among men), 62% had at least one prescription within two years prior to suicide. For deaths classified as undetermined intent, this corresponding number was 62% (86.0% among women and 55.1% among men) (Table 4). More than half (51.4%) of female deaths classified as undetermined intent had a prescription during the last month, compared to 32.8% among female suicides. However, during the last six months, 59.9% of female suicides and 65.4% of female deaths classified as undetermined intent had a prescription. Among males, 32.1% of suicides had a prescription during the last month compared to 18.1% of the deaths classified as undetermined intent.

**Table 4:** Elapsed time between last prescription and death, by sex and death mode

			**All methods**			
	**Women**		**Men**		**Total**	
Elapsed time since prescription*	**Suicide**	**Undetermined**	**Suicide**	**Undetermined**	**Suicide**	**Undetermined**
1 month	32.8 (30.6-35.0)	51.4 (47.0-55.8)	32.1 (30.7-33.5)	18.1 (15.7-20.5)	32.3 (31.6-33.0)	28.7 (27.6-29.8)
6 months	27.1 (25.0-29.2)	14.0 (10.9-17.1)	15.0 (13.9-16.1)	22.1 (19.6-24.6)	18.4 (17.7-19.1)	19.4 (18.3-20.5)
1 year	8.3 (7.0-9.6)	2.8 (1.3-4.3)	4.5 (3.9-5.1)	7.6 (6.0-9.2)	5.6 (5.0-6.2)	6.0 (5.4-6.6)
2 years	6.9 (5.7-8.1)	17.8 (14.4-21.2)	8.4 (7.6-9.2)	7.4 (5.8-9.0)	8.0 (7.0-5.9)	10.8 (9.9-11.9)
No prescription	24.9 (22.8-27.0)	14.0 (10.9-17.1)	40.0 (38.5-41.5)	44.9 (41.8-48.0)	35.7 (35.1-36.3)	34.8 (34.2-35.4)
			**Poisonings**			
	**Women**			**Men**		
Elapsed time since prescription*	**Suicide**	**Undetermined**	**Unintentional**	**Suicide**	**Undetermined**	**Unintentional**
1 month	40.6 (37.1-44.1)	36.8 (32.1-41.5)	27.5 (23.9-31.1)	31.9 (28.8-35.0)	24.7 (21.5-27.9)	18.8 (17.0-20.6)
6 months	32.7 (29.4-36.0)	25.3 (21.0-29.6)	28.2 (24.6-31.8)	28.5 (25.5-31.5)	25.8 (22.6-29.0)	22.5 (20.6-24.4)
1 year	8.7 (6.7-10.7)	7.0 (4.5-9.5)	9.4 (7.0-11.8)	9.0 (7.1-10.9)	12.2 (9.8-14.6)	12.5 (11.0-14.0)
2 years	4.3 (2.9-5.7)	8.8 (6.0-11.6)	11.4 (8.8-14.0)	5.9 (4.4-7.4)	9.4 (7.3-11.5)	10.6 (9.2-12.0)
No prescription	13.7 (11.3-16.1)	22.1 (18.0-26.2)	23.5 (20.1-26.9)	24.8 (22.0-27.6)	27.8 (24.5-31.1)	35.6 (33.4-37.8)

*Only available between 2006 and 2010.

In the restricted analysis on poisonings, suicides showed a higher percentage of prescribed drugs (86.3% among females and 75.2% among males). Deaths classified as undetermined intent and unintentional poisonings showed 77.9% and 76.5% among females and 75.2% and 64.4% among men. Further, when we calculated elapsed time between dispensation and death we found a gradient showing a prescription during the last six months on life for 73.3% of female suicides, 62.1% for female deaths classified as undetermined intent, and 55.7% for female unintentional poisonings. Similar figures for men were 60.4% for suicides, 51.5% for deaths classified as undetermined intent, and 41.3% among unintentional poisonings. There were no distinct differences in the use of neuroleptics, sedatives, soporifics and sedatives, SSRIs, or other antidepressants prior to suicide (data not shown).

Our multivariate analysis showed male deaths to more likely be classified as suicide than female deaths: OR: 1.13 (1.07-1.18). The youngest age group was also more likely to be classified as suicide. The risk of a death being classified as suicide increased for all time periods following 1987–1992. People born outside of Sweden had an increased risk of their deaths being classified as undetermined intent. Finally, divorced, unmarried, and widows/widowers had lower risks of their death being classified as suicide than married individuals: OR: 0.50 (0.47-0.53), OR: 0.52 (0.48-0.56), OR: 0.70 (0.63-0.77), respectively (Table 5).

**Table 5:** Odds ratios with 95% confidence intervals (CI) for death being classified as suicide compared to undetermined intent, by demographic background variables

**Variable**	**Odds ratios (95% CI)**
**Sex**	
Women	Ref
Men	1.13 (1.07-1.18)
**Age group**	
15-24	Ref
25-44	0.73 (0.66-0.80)
45-64	0.61 (0.56-0.67)
65+	0.89 (0.80-0.98)
**Time period**	
1987-1992	Ref
1993-1999	1.17 (1.10-1.25)
2000-2005	1.42 (1.32-1.53)
2006-2011	1.34 (1.25-1.44)
**Country of birth**	
Sweden	Ref
Other	0.77 (0.72-0.82)
**Marital status**	
Married	Ref
Divorced	0.50 (0.47-0.53)
Unmarried	0.52 (0.48-0.56)
Widow	0.70 (0.63-0.77)

## Discussion

This population-based study showed differences in marital status, educational level, country of birth, previous hospitalization for self-inflicted harm and substance abuse, as well as for prior psychiatric inpatient care and use of psychotropics between suicides and deaths classified as undetermined intent. Despite the differences in background variables, this information does not seem to be enough to establish guidelines on what share of deaths classified as undetermined intent should be interpreted as suicides.

However, our multivariate analysis showed the probability of a death being classified as suicide was increased for the following: males, the youngest individuals aged 15–24, deaths after 1987–1992, being born in Sweden, and being married. The strengths of the study include the nationwide Causes of Death Register with high coverage and completeness, together with linkage of numerous background factors. Sweden has a long tradition of routinely collecting nationwide data and the background variables we use are obtained from such registers with high quality and validity.

If it is not possible to distinguish between deaths classified as undetermined intent and deaths classified as suicide using high validity population-based registers in Sweden, our belief is that it is most likely not possible in other settings either. Another important conclusion based on our findings is that we suggest for future research to perform separate analyses in addition to the combined analyses of the two manners of death.

One important aspect is if the observed differences in background information are used in a prejudiced manner to purposely classify certain cases as suicides. However, this paper leaves that question unanswered.

Suicide studies usually either treat all deaths classified as undetermined intent as suicides or exclude them. However, both approaches are probably flawed and miscount the number [7]. The complex phenomenon that suicide represents with regard to religion and cultural and social norms may, without explicit evidence of suicidal intent, decrease the likelihood that a death is classified as suicide, and more so in some cultures and eras. Since the definition of deaths classified as undetermined intent is understood as they belong to either suicides or accidents, it is fair to say that a part of these deaths are in fact suicides [3,8,9]. However, despite this, it is difficult to quantify the proportion that are in fact suicides and those that are unintentional accidents. A study using Finnish data suggested that suicide mortality may be underestimated by 10%, indicating that around 30% of those deaths classified as undetermined intent actually were suicides [8]. Transferred to the Swedish population, this would give 30 more female suicide victims and 57 male suicide victims annually according to cause of death data from 2011.

During the observation period the mortality from undetermined intent in Sweden constituted around one-third of the suicide mortality among women and around one-fourth among men. The youngest women, aged 15–24, showed the lowest ratio of deaths due to undetermined intent, whereas among men the oldest, aged 65+, had the lowest ratio. Both of these groups displayed a lower proportion of poisonings among deaths classified as undetermined intent compared to the other age groups. If this lower proportion of poisonings explains or partly explains this finding is however not known. What is known is that poisoning is most difficult to classify according to manner of death. A British study reviewed all cases coded as either suicide or undetermined intent held at the Coroner’s Court of Birmingham and Solihull between January 1995 and December 1999 [2]. They found the elderly to have different characteristics and attributes from those at younger ages but didn’t say anything about the distribution of deaths classified as undetermined intent. The finding that male deaths were more likely to be classified as suicide than female is interesting considering the fact that men to a higher degree are involved in violent deaths, so the opposite therefore could be expected [20].

We know from previous studies that there is a social gradient in suicides [21]. In our study we compare amongst educational levels. If we had not found a social gradient in deaths classified as undetermined intents, that would have indicated these deaths were not suicides. However, our results did not let us draw any such conclusions despite our large cohort with unique high-quality data. Neither do our results, despite statistically significant differences in most background factors, allow us to establish a certain percentage of deaths classified as undetermined intent that should be regarded or interpreted as suicides.

An Italian study on educational level and mortality found suicide victims to have a higher education attainment among both females and males between the ages 15 to 64 compared with the same sex and age counterparts who died from natural causes [22]. In our study where we looked at all ages we found the opposite to be true, i.e., it was more common among suicide victims to have a lower level of education.

A major well-known risk factor for suicide is psychiatric disease [21,23]. The present study indicates some differences between suicide and deaths coded as undetermined intent regarding earlier psychiatric hospitalization. However, previous hospitalization for substance abuse was more common among deaths classified as undetermined intents. This is in contrary to an earlier study by Linsley where no significant differences in previous psychiatric treatment between the two manners of death were found [7]. Further, when we analyzed data on previous prescription of antidepressants, antipsychotics, and sedatives we found a tendency to a gradient where prescriptions were most common among suicides. Both the suicide trend and the trend for undetermined intent are similar with a relative decrease around 40-50% during the studied period. While several explanations have been given for the decrease in suicides (better awareness of health care personnel, different kinds of prevention programs, and a rise in the use of antidepressants) the reason for the decline in deaths classified as undetermined intents needs further investigation [24-26]. However, it would be reasonable to speculate that the decrease in deaths classified as undetermined intent naturally should follow the decline in suicides since a certain amount of suicides are misclassified as undetermined intent. We conclude that possible, yet unknown, changes in both random and nonrandom misclassification over time do not seem to affect suicide trends as shown by our data. In the analysis of poisonings we found hospitalization for substance abuse to be more frequent among deaths classified as undetermined intent and unintentional poisonings, which could indicate more uncertainty among deaths in known drug abusers with overdose, which is in line with the results of an American study from 2006 [15]. We included poisoning as both the underlying cause as well as the contributing cause to avoid differences in trends due to changes in the classification rules. The downside is that we obtain too many unintentional poisonings because all drug-related deaths are included (except alcohol). This means the level is too high, but the trend was more accurate and easier to interpret.

Our results were ambiguous where the time trends followed each other, which could be seen as actual suicides are a part of undetermined intents. The two manners of death differed in terms of most background factors, which might be interpreted as some of the deaths classified as undetermined intents are in fact accidents.

By analyzing Sweden’s unique high-validity population-based register data we found several differences in background variables between deaths classified as suicide and deaths classified as undetermined intent. However, we were not able to clearly distinguish these two manners of death. For future research we suggest separate analyses of the two different manners of death.

## Competing interests

The authors declare that they have no competing interests.

## Authors’ contributions

AF, LAJ, IT originated the idea, CB, RL, IT designed the study, CB performed the data analysis and wrote the manuscript draft, all authors contributed in analyzing the data and in the completing of the manuscript.

## References

[B1] Varnik P Sisask M Varnik A Yur'yev A Kolves K Leppik L Nemtsov A Wasserman D Massive increase in injury deaths of undetermined intent in ex-USSR Baltic and Slavic countries: hidden suicides? Scand J Public Health 2010 38 4 395 403 10.1177/1403494809354360 19933222

[B2] Tadros G Salib E Elderly suicide in primary care Int J Geriatric Psychiatry 2007 22 750 756 10.1002/gps.1734 17152111

[B3] O’Carroll PW A consideration of the validity and reliability of suicide mortality data Suicide Life Threat Behav 1989 19 1 16 2652382 10.1111/j.1943-278x.1989.tb00362.x

[B4] Allebeck P Allgulander C Henningsohn L Jakobsson SW Causes of death in a cohort of 50,465 young men–validity of recorded suicide as underlying cause of death Scand J Soc Med 1991 19 242 247 1775959 10.1177/140349489101900405

[B5] Connolly JF Cullen A Under-reporting of suicide in an Irish county Crisis 1995 16 34 38 7614831 10.1027/0227-5910.16.1.34

[B6] Kleck G Miscounting suicides Suicide Life Threat Behav 1988 18 219 236 3188138 10.1111/j.1943-278x.1988.tb00158.x

[B7] Linsley KR Schapira K Kelly TP Open verdict v. suicide - importance to research Br J Psychiatry 2001 178 465 468 10.1192/bjp.178.5.465 11331564

[B8] Ohberg A Lonnqvist J Suicides hidden among undetermined deaths Acta Psychiatr Scand 1998 98 214 218 10.1111/j.1600-0447.1998.tb10069.x 9761408

[B9] Phillips DP Ruth TE Adequacy of official suicide statistics for scientific research and public policy Suicide Life Threat Behav 1993 23 307 319 8310465

[B10] Pritchard C Hean S Suicide and undetermined deaths among youths and young adults in latin America: comparison with the 10 major developed countries–a source of hidden suicides? Crisis 2008 29 145 153 18714911 10.1027/0227-5910.29.3.145

[B11] Speechley M Stavraky KM The adequacy of suicide statistics for use in epidemiology and public health Can J Public Health 1991 82 38 42 2009484

[B12] World Health Organization International Statistical Classification of Diseases and Related Health Problems, Tenth Revision 1992 Geneva: World Health Organization 1992 1994

[B13] Mohler B Earls F Trends in adolescent suicide: misclassification bias? Am J Public Health 2001 91 150 153 11189813 10.2105/ajph.91.1.150 PMC1446515

[B14] Rockett IR Hobbs G De Leo D Stack S Frost JL Ducatman AM Kapusta ND Walker RL Suicide and unintentional poisoning mortality trends in the United States, 1987–2006: two unrelated phenomena? BMC Public Health 2010 10 705 10.1186/1471-2458-10-705 21083903 PMC3091585

[B15] Hempstead K Manner of death and circumstances in fatal poisonings: evidence from New Jersey Inj Prev 2006 12 Suppl 2 ii44 ii48 17170171 10.1136/ip.2006.012583 PMC2563476

[B16] Schneider B Substance use disorders and risk for completed suicide Arch Suicide Res 2009 13 303 316 10.1080/13811110903263191 19813108

[B17] Stanistreet D Taylor S Jeffrey V Gabbay M Accident or suicide? Predictors of Coroners’ decisions in suicide and accident verdicts Med Sci Law 2001 41 111 115 11368390 10.1177/002580240104100205

[B18] Ludvigsson JF Otterblad-Olausson P Pettersson BU Ekbom A The Swedish personal identity number: possibilities and pitfalls in healthcare and medical research Eur J Epidemiol 2009 24 659 667 10.1007/s10654-009-9350-y 19504049 PMC2773709

[B19] Wettermark B Hammar N Fored CM Leimanis A Otterblad Olausson P Bergman U Persson I Sundstrom A Westerholm B Rosen M The new Swedish prescribed drug register–opportunities for pharmacoepidemiological research and experience from the first six months Pharmacoepidemiol Drug Saf 2007 16 726 735 10.1002/pds.1294 16897791

[B20] Karch DL Logan J McDaniel D Parks S Patel N Centers for Disease C, Prevention Surveillance for violent deaths–national violent death reporting system, 16 states, 2009 MMWR Surveill Summ 2012 61 1 43 22971797

[B21] Qin P Agerbo E Mortensen PB Suicide risk in relation to socioeconomic, demographic, psychiatric, and familial factors: a national register-based study of all suicides in Denmark, 1981–1997 Am J Psychiatry 2003 160 765 772 10.1176/appi.ajp.160.4.765 12668367

[B22] Pompili M Vichi M Qin P Innamorati M De Leo D Girardi P Does the level of education influence completed suicide? A nationwide register study J Affect Disord 2013 147 437 440 10.1016/j.jad.2012.08.046 23021379

[B23] Mortensen PB Agerbo E Erikson T Qin P Westergaard-Nielsen N Psychiatric illness and risk factors for suicide in Denmark Lancet 2000 355 9 12 10.1016/S0140-6736(99)06376-X 10615884

[B24] Bellanger MM Jourdain A Batt-Moillo A Might the decrease in the suicide rates in France be due to regional prevention programmes? Soc Sci Med 2007 65 431 441 10.1016/j.socscimed.2007.03.027 17475385

[B25] Gibbons RD Hur K Bhaumik DK Mann JJ The relationship between antidepressant medication use and rate of suicide Arch Gen Psychiatry 2005 62 165 172 10.1001/archpsyc.62.2.165 15699293

[B26] Korkeila J Salminen JK Hiekkanen H Salokangas RK Use of antidepressants and suicide rate in Finland: an ecological study J Clin Psychiatry 2007 68 505 511 10.4088/JCP.v68n0403 17474804

